# Supplementary material for: Enhancing Optical and Thermal Stability of Blue-Emitting Perovskite Nanocrystals through Surface Passivation with Sulfonate or Sulfonic Acid Ligands
Source: Nanomaterials (Basel). 2024 Jun 18;14(12):1049. doi: 10.3390/nano14121049 (PMC11206382; doi:10.3390/nano14121049)
Supplement: Supplementary file 1 [file nanomaterials-14-01049-s001.zip › nanomaterials-3052886-supplementary.pdf]

Supporting Information

Enhancing Optical and Thermal Stability of Blue-emitting Perovskite Nanocrystals through Surface Passivation with Sulfonate or Sulfonic Acid Ligands

Shu-Han Huang[1], Sheng-Hsiung Yang[1,*], Wen-Cheng Tsai[2] and Hsu-Cheng Hsu[2,3]

[1]*Institute of Lighting and Energy Photonics, College of Photonics, National Yang Ming Chiao Tung University, Tainan 711010, Taiwan*

[2]*Department of Photonics, National Cheng Kung University, Tainan 70101, Taiwan*

[3]*Program on Key Materials, Academy of Innovative Semiconductor and Sustainable Manufacturing, National Cheng Kung University, Tainan 70101, Taiwan*

*Correspondence: yangsh@nycu.edu.tw

Table S1. Carrier lifetime parameters of TR-PL decay curves of the original and modified perovskite NCs.

perovskite NCs	A1 (%)	τ_1 (ns)	A2 (%)	τ_2 (ns)	A3 (%)	τ_3 (ns)	τ_{avg} (ns)
original	74.13	1.91	24.35	8.24	1.52	50.14	17
SBS-modified	82.51	2.38	16.2	14.19	1.29	106.99	32.6
SbSS-modified	78.27	1.87	20.1	11.18	1.63	74.42	23.9
SPTS-modified	80.87	2.63	17.28	16.72	1.85	118.83	43.6
DBSA-modified	79.5	1.72	19.08	10.09	1.42	67.17	7.1

*τ_1, τ_2 and τ_3 are the lifetimes of different recombination decays.

The average lifetime was calculated by *Eq* (S1):

$$\tau_{avg} = \frac{A_1\tau_1^2 + A_2\tau_2^2 + A_3\tau_3^2}{A_1\tau_1 + A_2\tau_2 + A_2\tau_3} \qquad Eq(S1)$$

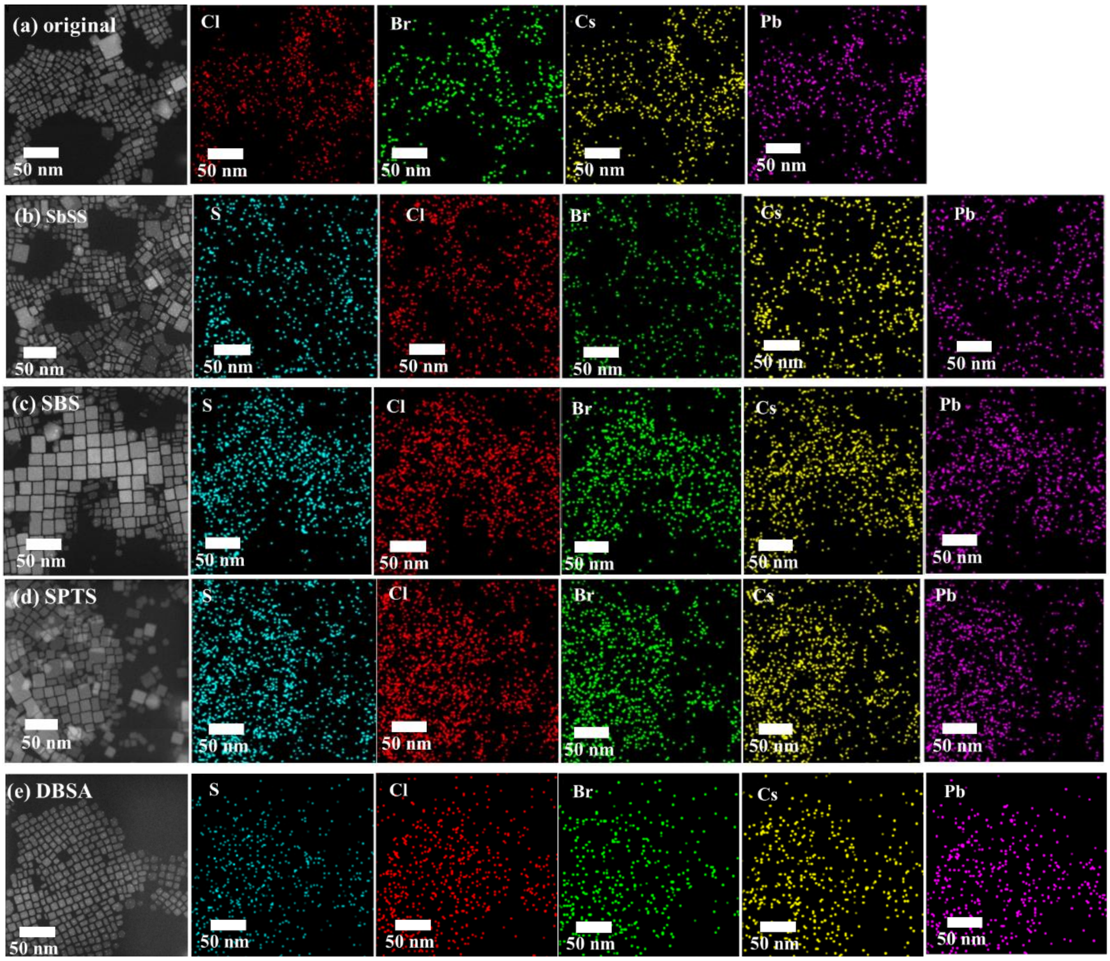

Figure S1. HAADF-STEM images and elemental mapping of S, Cl, Br, Cs, and Pb elements of the (a) original and (b) SbSS-, (c) SBS-, (d) SPTS-, (e) DBSA-modified perovskite NCs.

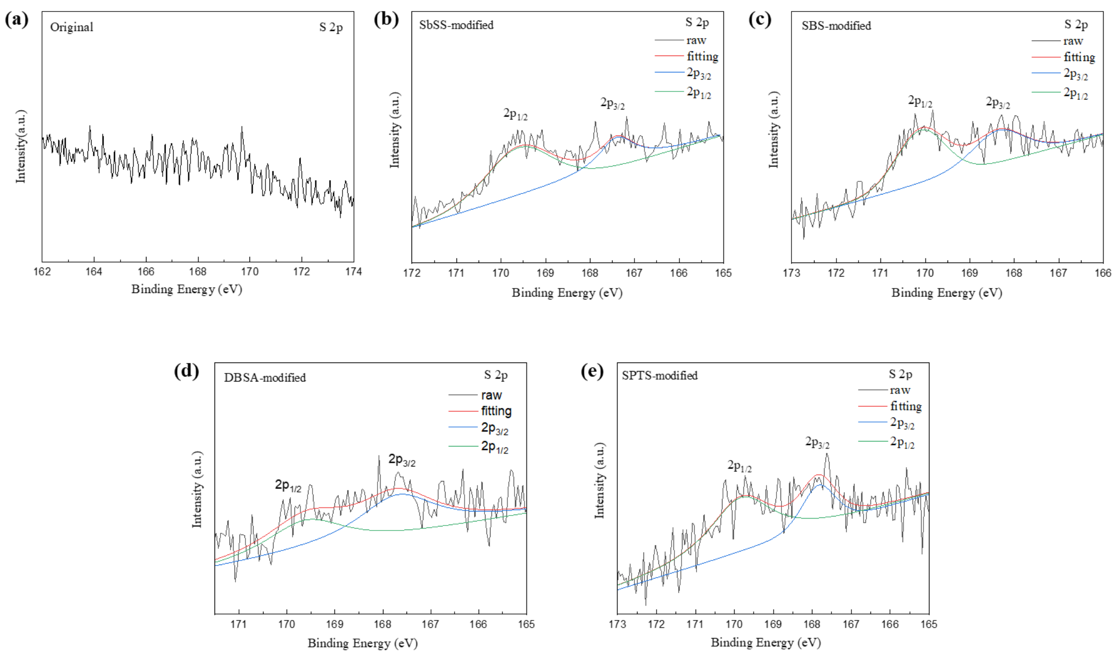

Figure S2. S 2p core-level XPS spectra of the (a) original and (b) SbSS-, (c) SBS-, (d) SPTS-, (e) DBSA-modified perovskite NCs.

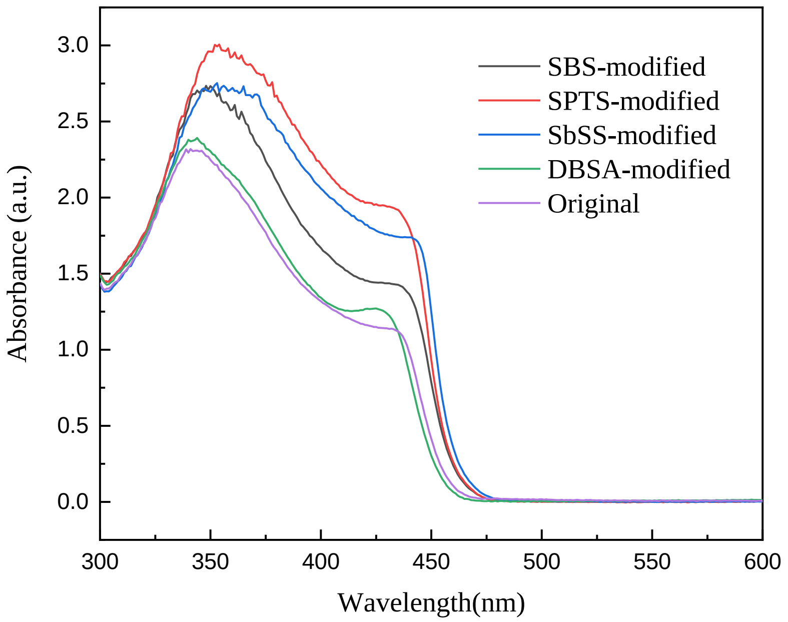

Figure S3. UV-vis absorption spectra of the original and sulfonate (or sulfonic acid) ligand-modified perovskite NCs.

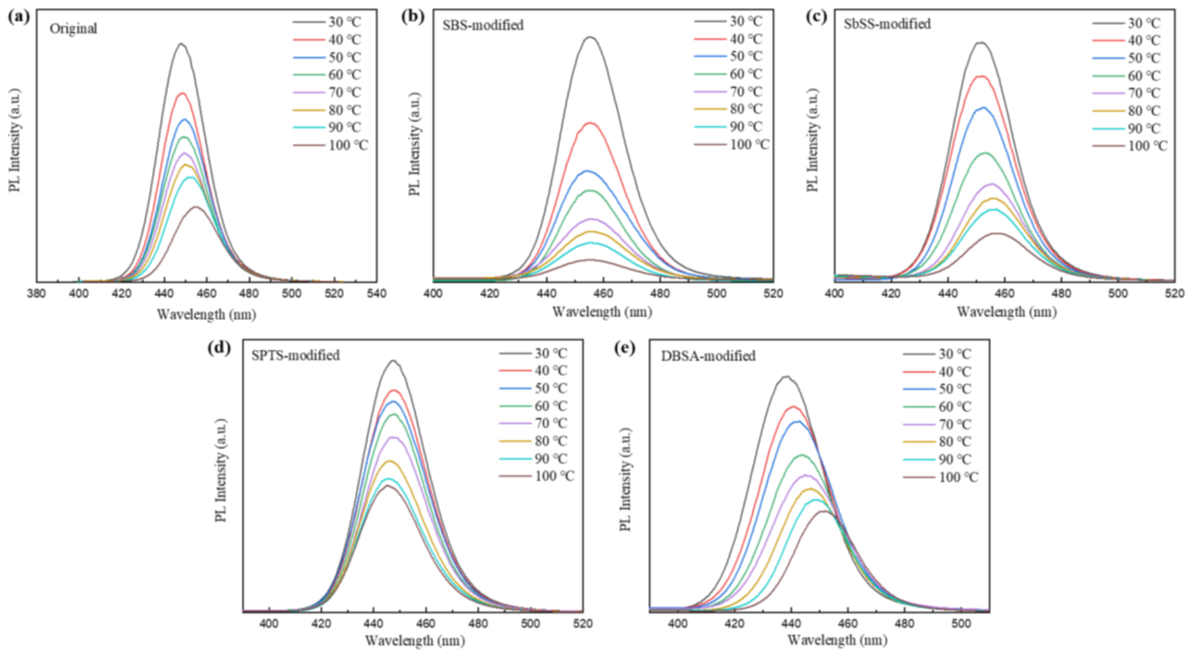

Figure S4. PL spectra of the (a) original and (b) SBS-, (c) SbSS-, (d) SPTS-, (e) DBSA-modified perovskite NCs at different heating temperatures.

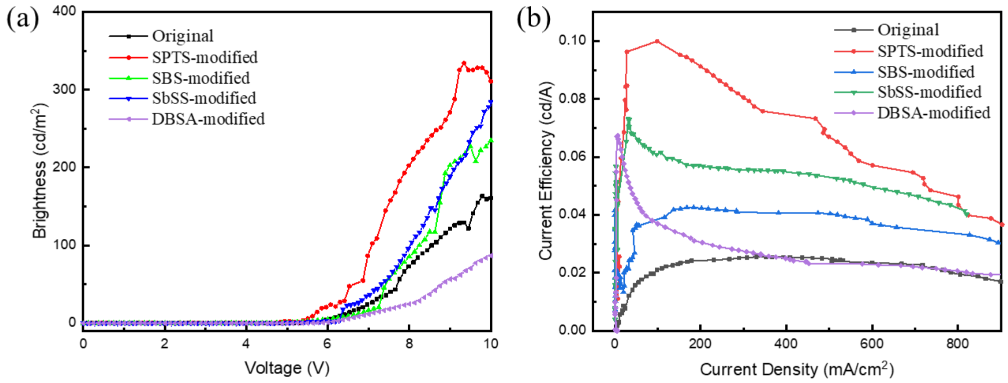

Figure S5. (a) Brightness–voltage and (b) current efficiency–current density characteristics of PeLEDs.
